# Supplementary material for: TRMT2B is responsible for both tRNA and rRNA m5U-methylation in human mitochondria
Source: RNA Biol. 2020 Jan 17;17(4):451–62. doi: 10.1080/15476286.2020.1712544 (PMC7237155; doi:10.1080/15476286.2020.1712544)
Supplement: Supplemental Material [file krnb-17-04-1712544-s001.pdf]

SUPPLEMENTAL FIGURES

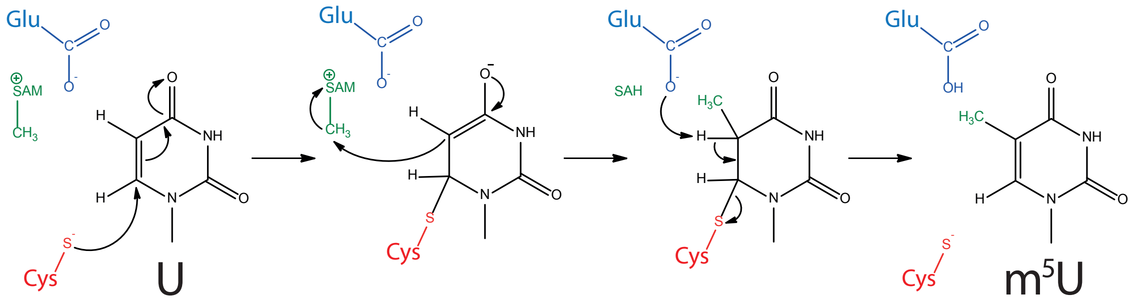

Figure S1: The catalytic mechanism of m^{5}U-methyltransferases, showing the involvement of the nucleophilic cysteine, the proton extracting glutamate, and S-adenosylmethionine (SAM).

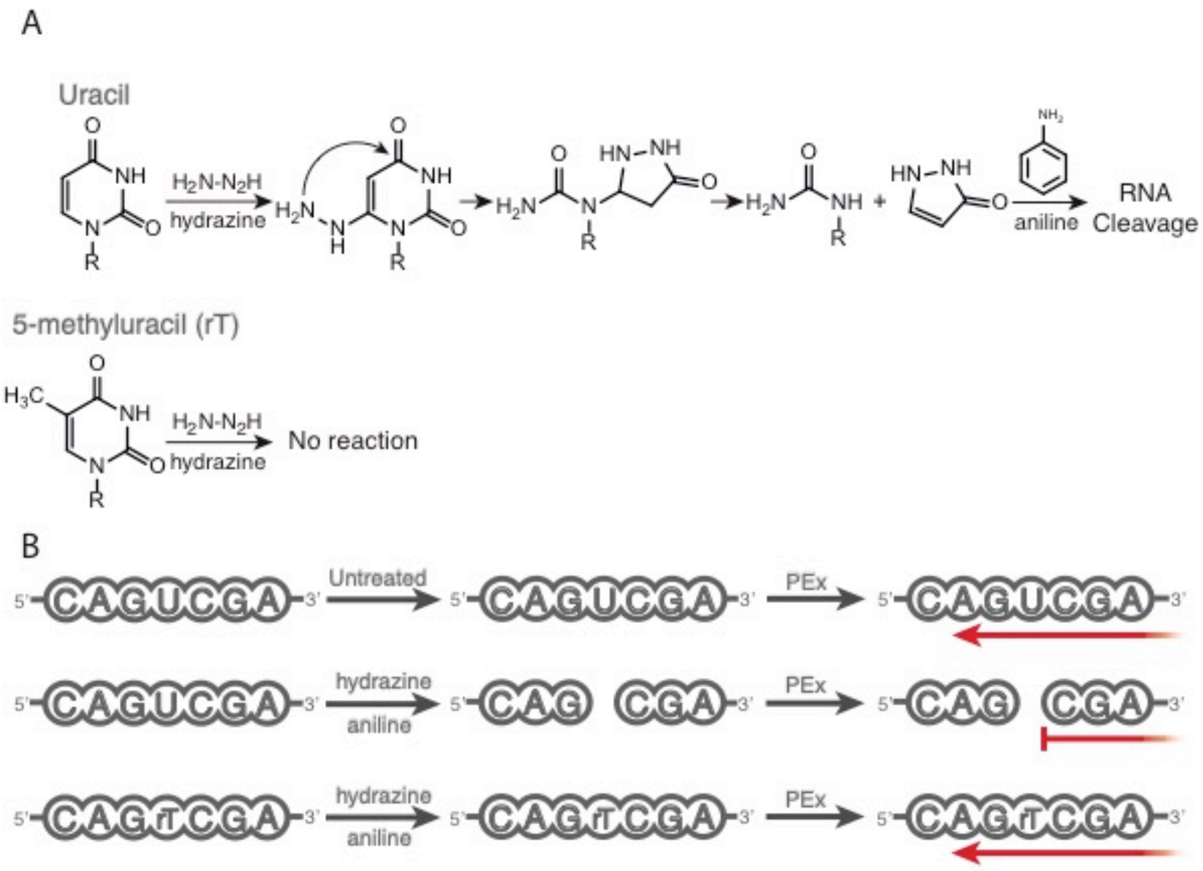

Figure S2: Detection of 5-methyluridine in RNA (a) The hydrazinolysis of uracil in RNA is mediated through the nucleophilic attack of C6 by hydrazine, and the subsequent reaction with C4, forming a pyrazole which is spontaneously cleaved from the glycosidic nitrogen. The resulting abasic site is susceptible to β-elimination at both the 3' and 5' phosphates in the presence of aniline. A methyl group at C5 protects the pyrimidine ring from nucleophilic attack, and therefore subsequent strand cleavage at this position **(b)** Primer extension (red arrow) following Hydrazine-Aniline treatment will result in stalled extension products corresponding to unmodified uracils in the original sequence, whereas primers will read through m^5U. These products are then separated through gel electrophoresis.

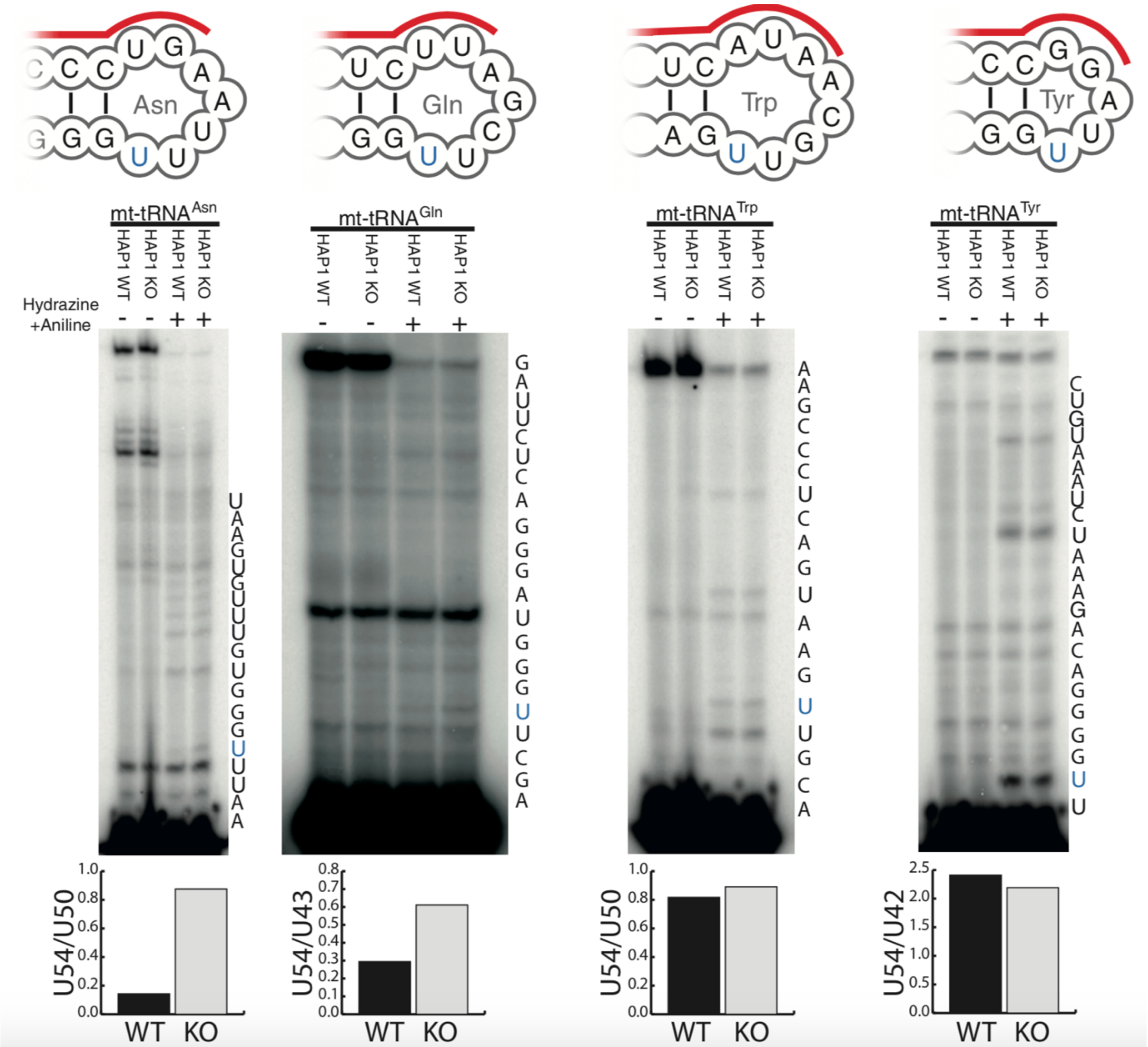

Figure S3: Identification of TRMT2B catalysed m^{5}U54 in mt-tRNAs. RT-PEx reactions performed and quantified as in **Figure 3** using [^{32}P]-end labelled primers complementary to the corresponding sequences in mt-tRNA^{Asn}, mt-tRNA^{Gln}, mt-tRNA^{Trp}, and mt-tRNA^{Tyr}.

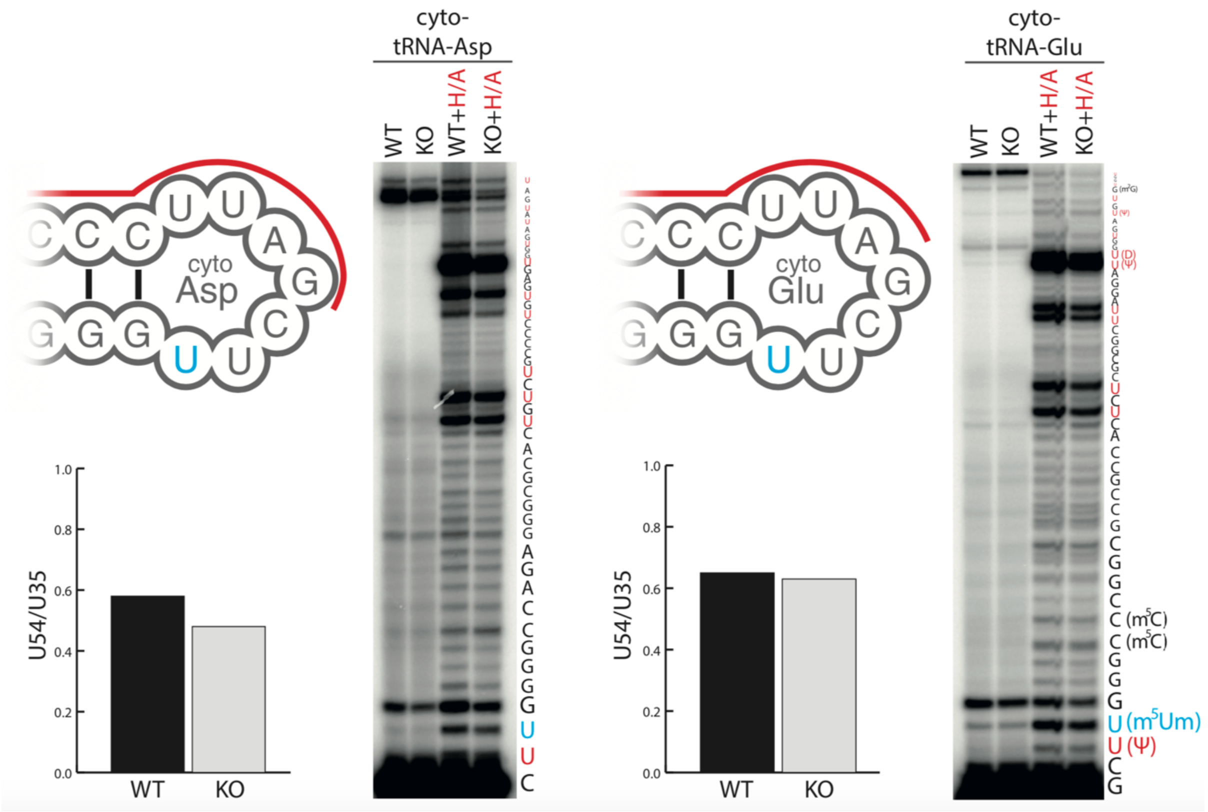

Figure S4: Absence of TRMT2B catalysed m^{5}U54 in cyto-tRNAs. RT-PEx reactions performed and quantified as in **Figure 3** using [^{32}P]-end labelled primers complementary to the corresponding sequences in cyto-tRNA^{Asp} and cyto-tRNA^{Glu}.
